# Flying fruit flies correct for visual sideslip depending on relative speed of forward optic flow

**DOI:** 10.3389/fnbeh.2013.00076

**Published:** 2013-07-02

**Authors:** Stephanie Cabrera, Jamie C. Theobald

**Affiliations:** Department of Biological Science, Florida International UniversityMiami, FL, USA

**Keywords:** *Drosophila*, vision, parallax, optomotor response, depth cue

## Abstract

As a fly flies through its environment, static objects produce moving images on its retina, and this optic flow is essential for steering and course corrections. Different types of rotation and translation produce unique flow fields, which fly brains are wired to identify. However, a feature of optic flow unique to translational motion is that adjacent images may move across the retina at different speeds, depending on their distance from the observer. Many insects take advantage of this depth cue, called motion parallax, to determine the distance to objects. We wanted to know if differential object speeds affect the corrective responses of fruit flies when they experience unplanned course deviations. We presented tethered flying flies with optic flow and measured their corrective responses to sideways perturbations of images with different relative forward speeds. We found that flying flies attend to the relative speed of dots during forward motion, and adjust their corrective responses to sideslip deviations depending on this cue. With no other distinguishing features (such as brightness or size), flies mounted a greater response to sideways deviations that were signaled by faster moving dots in the forward flow field, those that appeared radially closer by their speeds. This is consistent with the interpretation that fruit flies attend to seemingly nearer objects, and correct more strongly when they indicate a perturbation.

## Introduction

For a flying insect, detecting deviations from course, and correcting them, is essential for tracking down resources, and an important jobs of its visual system (Egelhaaf and Kern, 2002). However, eyes focus light from the three dimensional world onto a two dimensional plane, which can image both near and far objects onto similar positions on the retina (Land and Nilsson, 2002). To reconstruct the three dimensional environment, animals often rely on depth cues: aspects of a scene that provide information about the real distance to objects (Howard, 2002). We set out to determine how fruit flies use translational velocity, a motion parallax depth cue, while correcting for course perturbations during flight.

Many insects have specialized eyes and brain regions to accommodate their flight abilities (Egelhaaf and Kern, 2002). They detect motion in small receptive fields (Eichner et al., 2011), then integrate these over the visual field to extract potentially important patterns, such as looming collisions (Wicklein and Strausfeld, 2000; Gabbiani et al., 2002; Rind and Santer, 2004), targets to intercept (O'Carroll, 1993; Geurten et al., 2007), or their own motion relative to the world (Hausen and Egelhaaf, 1989; Krapp and Hengstenberg, 1996; Franz and Krapp, 2000). Determining self-motion is particularly important to small flying animals, as even tiny air currents might push or turn them in ways that destabilize their flight (Combes and Dudley, 2009). To compensate for this, insects have robust corrective responses that restore their heading when they perceive they've deviated from it with motion they didn't initiate (Collett, 1980a; Egelhaaf et al., 1988; Mronz and Lehmann, 2008; Theobald et al., 2010a).

Visually detecting self-motion depends on surrounding objects, but distance is visually ambiguous-a nearby flower might occupy the same space on the retina as the moon. Depth cues can resolve this problem (Howard, 2002), but insects have some limitations. First, their rigid bodies leave the eyes in fixed position and focus, so they cannot find depth by visual accommodation or convergence (Srinivasan, 1992). Second, insects are small, and although several possess and use binocular vision (Beersma et al., 1977), their eyes are necessarily close together. This limits the possible use of binocular stereopsis to objects no more than a few centimeters off (Collett, 1987), and only a few groups, such as mantids, have been convincingly shown to use this mechanism (Rossel, 1983, 1986; Eriksson, 1985).

For moving insects an excellent alternative is motion parallax (Srinivasan, 1992). This depth cue is related to stereopsis, but instead of comparing displaced images from each eye, motion parallax relies on the motion of the animal itself for displacement. Translational self-motion through a stationary environment generates images that follow characteristic field lines (Gibson, 1950; Koenderink, 1986), with a speed on the retina proportional to: the viewer's actual speed, the sine of their angle from the forward direction, and the inverse of their distance (Horridge, 1987; Srinivasan, 1992). The inverse distance relationship allows us, when we walk, to determine that tree branches whizzing by are close, but the hovering moon is far off.

Several insects use this cue. Locusts face a target and sway their heads from side-to-side (called “peering”) right before a jump (Wallace, 1959). This generates translational self-motion which signals distance, and locusts adjust their jump force accordingly (Sobel, 1990a,b; Kral and Poteser, 1997). Flying honey bees judge object distances during flight by image velocities (Kirchner and Srinivasan, 1989; Srinivasan et al., 1991; Dacke and Srinivasan, 2007). Many bees and wasps learn their nest positions by performing orientation flights upon exit (Wehner, 1981), in which they turn back and face the nest while flying in arcs, a motion that reveals the distance of nearby objects and aids in the return flight (Zeil, 1993). Walking fruit flies select objects to approach based on apparent nearness (Götz, 1994), using image displacement generated by their own translational motion (Schuster et al., 2002). During flight, flies steer, and move their heads in ways that can minimize rotational components of the optic flow field. This isolates the translational components which in turn provide information about their three dimensional surroundings (Schilstra and van Hateren, 1998; Krapp, 2000).

When image trajectories indicate a fly has gone off course, it generates corrective steering. Distance may be a useful factor in optimizing this control effort. To determine if flies use parallax cues during corrective steering, we presented optic flow, with depth cues, simulating forward motion and sideways deviations. We speculated flies might respond in one of three ways. First, if seemingly near and far objects underwent similar sideways motion, flies might respond identically, indicating they do not use parallax from forward motion for sideways corrective responses. Second, flies might respond more strongly to seemingly near objects, since near objects constitute their close surroundings. Third, flies might respond more strongly to seemingly distant objects, since during translation distant objects appear to move relatively little, and perturbations imply larger course deviations than the same image motion from nearby objects.

## Materials and methods

### Experimental subjects

We collected female *Drosophila melanogaster* (Meigen) wild-type, Oregon-R strain, 3–6 days after adult eclosion. The colony followed a natural light cycle in bottles on a standard medium. We cold-anesthetized and then tethered the flies by gluing a rigid tungsten rod, 0.02 mm diameter, to the dorsal prothorax. After about an hour for recovery flies could flap their wings without interference from the rod. We then affixed the tethered fly into the center of the flight arena, where each performed every trial of an experiment without repetition. We included data from every fly that beat its wings for the duration of the experiment.

### Visual stimulation

**Figure 1:**
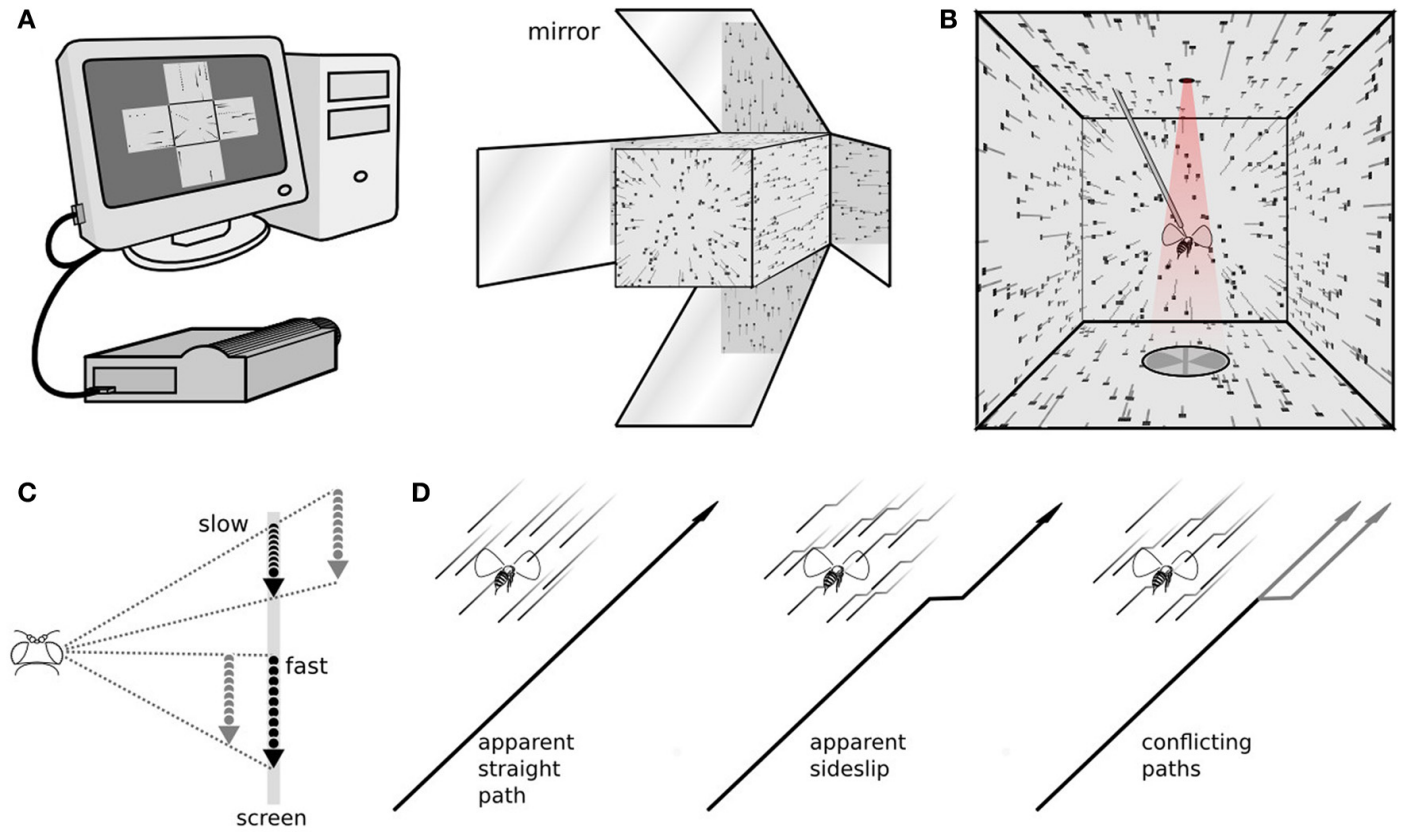
**The stimulus and response measurements. (A)** A computer with a dedicated graphics processing unit connects to a high frame rate projector, viewed here from the front, which projects images of a three dimensional scene on to five faces of a cube. **(B)** The fly, viewed from the back, is rigidly tethered in the center of the cube. From this vantage point the images on the walls appear as a perspective-corrected three dimensional scene, here showing dots, at the size and density used in our experiments, flowing from the front to back behind the fly. The trailing gray lines illustrate the dot velocities, but weren't part of the stimulus. Above the fly is an infrared light, and below are a pair of photodiodes, that together measure the amplitude of each wingstroke. **(C)** Dots move at different speeds across the screen. A moving viewer can reconcile these as stationary points at different distances. **(D)** Optic flow simulating forward motion, forward motion with an abrupt sideslip, and an ambiguous situation, where some points indicate a straight path and others a sideways deviation.

Our flight arena was a perspex cube, with edges measuring 200 mm, and an open side in the back. The five remaining sides had back projection screen material (DAL41468 Da-Lite Da-Tex Rear) covering their inner surfaces. With first-surface mirrors affixed at the back and angled 45 degrees to the sides, a projector in front can illuminate all five sides at once, covering 10.47 steradians of the visual field (Figure 1A). The projector (Lightspeed Designs DepthQ 360) imaged a different view of a three dimensional scene onto each face of the cube (four of them reversed to account for the mirror reflections), and updated at 360 frames per second. Each view was perspective corrected for an observer located in the center of the cube (Figure 1B). Our customized software interacted directly with a dedicated graphics card using the OpenGL API. The resolution on the front face was 229 × 229 pixels, or 2.5 pixels/degree from the perspective of the fly. On the sides the resolution was 200 × 200, or 2.2 pixels/degree. The small difference is due to the shorter path between the projector and front surface, which then gets an image of the same size but at a higher pixel density. To maximize the contrast of the projector based display, we turned off the room lights during experiments, which produced a measured contrast of 98% between dots and background (measured with a Gossen Starlight 2 lightmeter).

Each experiment consisted of multiple open loop trials interspersed by short bouts of closed loop stripe fixation. During stripe fixation the fly's wing beats control the position of a rotating vertical bar. It is not part of the analyzed experiments, but stripe fixation between experiments increases the length of time a fly will continue responding to visual stimulation, and also helps assure each fly is in a similar behavioral state immediately before each trial, actively controlling flight (Reichardt and Wenking, 1969; Heisenberg and Wolf, 1979). During experimental trials the vertical bar disappeared and a field of randomly placed dots appeared. Each dot was 3 pixels wide, or just over a degree of visual angle, smaller than the ~5° interommatidial angle of a fly (Heisenberg and Wolf, 1984). These dots then flowed around the fly to simulate forward translational motion (Figure 1C), and slip sideways depending on the particular experiment and trial (Figure 1D). Previously we have found flowing dots to be a powerful visual stimulus to evoke steering responses while simulating almost any kind of motion for the fly (Theobald et al., 2010a). Those experiments took place in a cylindrical LED arena, but the dot motion here is identical. Each dot was imaged on the screen whenever it was a certain virtual distance from the fly. During simulated translation, dots that came into viewing range appeared on the screen and moved according to the fly's perspective, and dots that left the viewing range disappeared, which allowed us to maintain constant mean dot density in all directions, about 89 dots per steradian. Dots make the transition from one screen to another fairly seamlessly, for example leaving the front screen and appearing on the left. (see Supplementary video).

To simulate forward translation for the fly, dots appeared in the forward visual region near the focus of expansion, moved around the fly above, below, and to the sides, and began to converge behind the fly. The rear panel of the cube is omitted to make room for tethering, so the focus of contraction, in the rearmost region, was not imaged. We simulated sideways motion by superimposing onto this forward dot motion a lateral focus of expansion, to the left or right. Because these are translational movements and dots are assigned random locations in space, adjacent dots move in the same direction but possibly at different speeds (Figure 1C). This is motion parallax, the different image speeds that can convey object distance. The image speeds also depend on the location in the flow field, and the image of a close object near the focus of expansion can move more slowly than the image of a far off object that is perpendicular to the focus of expansion. However, at any specific position in the flow field, when an animal translates, the images of physically nearer objects will appear to move faster. Only dot speed indicated radial distance. No other cues, such as size or brightness, were enabled here.

### Steering responses

We measured the behavioral effects of our visual presentations by using a wing beat analyzer to determine the differences in left and right wing stroke amplitude for each wing beat. An infrared light positioned above the fly cast a shadow of its wings onto a pair of photodiodes positioned below. The wing beat analyzer measures the occlusion of light for each wing during each stroke (about 200 beats per second), and larger beats block more of the light. This technique does not capture the full three dimensional dynamics of the wing stroke, but the difference between the left and right wing beat amplitude signal (ΔWBA) is proportional to yaw torque (Götz, 1987; Frye and Dickinson, 2004). Between experimental trials, during the closed loop bouts of stripe fixation, the ΔWBA signal fed back to the display computer to update the stripe position, and an active healthy fly actively steers to keep the bar approximately in front. This helps keep the fly engaged in experiments. During the open loop experimental trials, the ΔWBA signal was measured along with timing pulses from the stimulus generator, to ensure fly responses were correctly aligned in time with the stimuli they viewed.

## Results

### Response to an abrupt slip

**Figure 2:**
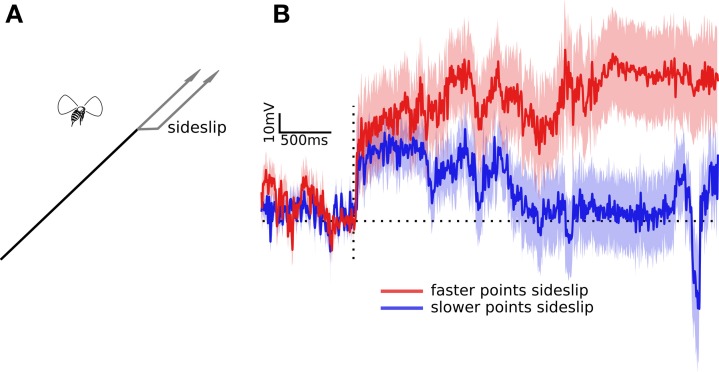
**Wing beat responses to two partial impulses in sideslip. (A)** Optic flow of dots simulates two conflicting paths, some dots indicate forward motion, others indicate forward motion with an abrupt sideways deviation. The actual experimental perturbations were randomly to the left or right. **(B)** The traces illustrates the difference between the left and right wing beat amplitudes. The red trace shows the response to the faster dots (apparently nearer) deviating to the side, while the blue trace is the response to the slower dots (apparently farther) deviating to the side. Traces are the means flanked by standard errors from the responses of 100 flies. The horizontal dotted line shows the response level when left and right wing beat amplitude are equal, the vertical dotted line is the time point of the lateral deviation.

In previous experiments we displayed an entire flow field of dots moving together, simulating perspective corrected translational or rotational motion (Theobald et al., 2010a,b). Dots were perturbed along perspective corrected flow lines and flies compensated with wing beat responses that would tend to stabilize their position against the perceived motion. The dots represented small objects or features in random directions and at random distances. For rotational motion distance has no effect, but during translation, the virtual distances can cause adjacent dots to move at different speeds. To determine if translating fruit flies attend to these different object speeds for corrective responses, we presented a stimulus that began just as previous experiments, with a field of dots simulating forward optic flow and an abrupt slip to the left or right. This elicits a reliable corrective wing beat response from the fly. However we presented conflicting stimuli by only sideslipping the dots that had been previously moving quickly (simulated to represent nearer objects) or the dots that had been moving slowly (simulated to represent more distant objects). Speed varied as dots moved through the flow field, and every dot was slower near the focus of expansion and faster near perpendicular to the axis of motion, but dots that represented closer objects moved proportionally more quickly at each location. Aside from speed during forward translation the dots were otherwise identical; they were the same size, brightness, and slipped sideways by the same amount (Figures 1D, 2A). In other words, their relative speed in the flow field was the only cue to their distance.

As in previous experiments flies compensated for sideslip by adjusting their relative wing stroke amplitudes. Within tens of milliseconds they increased the wing stroke amplitude of one wing, while decreasing the other, as if to follow the frontal slip of the dots. But in this case both the amplitude and time course of the response were affected by which of set of dots had slipped. Figure 2B shows the mean responses of 100 flies after a visual stimulus simulating forward motion and an abrupt slip to the side. The red trace shows the wing beat response to the faster dots, those simulating nearer objects, side slipping, and the blue trace shows the response to the slower dots, those simulating farther objects, slipping. In each case the dots that didn't slip indicated a steady forward course, so the left and right wing beat responses could only be produced by the sideslipping dots. Again, these dots only differed in speed of forward motion, they sideslipped at the same speed by the same amount. However they produced different responses. The delay times of the initial response, estimated as the time at which the responses were more than a standard error above zero, were 33 ms for the faster moving dots, and 46 ms for the slower moving dots. The time to peak responses were difficult to estimate as the conflicting flow fields in these experiments produced weak and noisy wing beat responses. However, the response to the faster moving dots is consistently higher, and diverges by more than a standard deviation from the response to the slower moving dots at 734 ms. The faster response also agreed with previous results in that the response didn't quickly return to baseline, requiring more than the three seconds that we measured before the mean wing beat amplitudes were equal. The slower response was back to one standard error of baseline within 1703 ms.

### Linear dynamical responses to pseudorandom white noise

**Figure 3:**
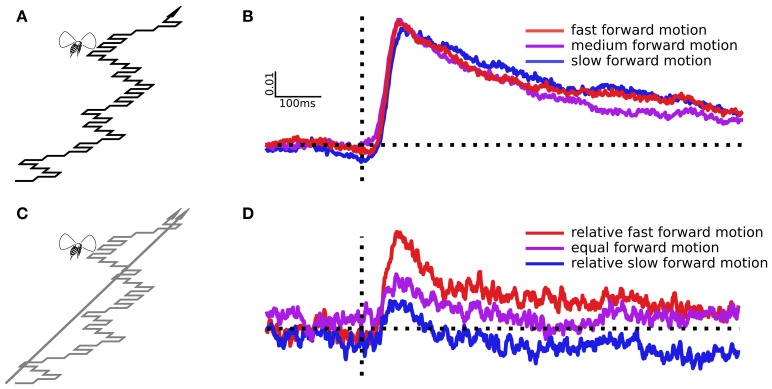
**Sideslip following a pseudo white noise sequence and the resulting impulse responses. (A)** The virtual path moves forward, but also slips left or right in every frame according to a binary m-sequence, part of which is illustrated here. **(B)** Impulse responses estimated from virtual paths with three different forward speeds, but identical side-to-side motion. **(C)** Here the dots were split, half simulating a straight ahead trajectory, half simulating sideslip following an m-sequence. **(D)** Impulse responses from the slipping points, which moved identically in each trial, but in the presence of three different straight ahead dot speeds, which change the relative forward speed of the slipping points.

Characterizing the response to visual slip by simulating a single event repeatedly is time consuming, and can produce noisy results. A useful alternative is to move the points continuously, modulated by a white noise sequence, and then cross-correlate this sequence with the response. This extracts the dynamic linear filter that best predicts the responses to the input sequence, which in a linear system is the impulse response (Ringach and Shapley, 2004). Importantly, it reveals the time course of an ideal, linear response to motion and allows us to predict how tracking would occur under more natural circumstances. To determine the effect of forward speed on the dynamical responses to sideslip, we followed the protocols of previous experiments (Theobald et al., 2010a,b), but modified for a new visual arena. We modulated side-to-side dot motion with a pseudorandom binary series called a maximal length sequence, or m-sequence (Golomb, 1981). These sequences are spectrally flat, have near zero autocorrelation, and contain every possible sequence of left and right deviations, up to the order of the sequence. This makes them a compact way to discern responses at many temporal frequencies. We began with dots that moved coherently, all simulating a forward path with abrupt slips, updated 360 times per second. On every 3rd frame (120 times per second) the scene sideslipped to the right or left, modulated with a 10th order binary m-sequence (Figure 3A). We used three forward translational speeds to simulate slow, medium, or fast forward motion, corresponding to objects that appear increasingly nearby. The apparent forward speed could be characterized in different ways, since the speed and direction of dots depends on their angle from the focus of expansion, and their virtual distance from the observer. But dots move fastest when they are perpendicular to the direction of travel, and we set the mean angular speed of dots perpendicular to the direction of travel, (to the sides, bottom, or top) to either 34°/s (slow), 69°/s (medium) or 138°/s (fast). How far away these objects seem depends on how fast the observer believes itself to be moving, but for a typical fly moving at 0.5 meters per second, they correspond objects at distances of 0.84, 0.42, and 0.21 m, with the faster moving images corresponding to seemingly closer objects.

We recorded the ΔWBA from 61 flies during white noise stimulation. Impulse responses to sideways deviations are largely unaffected by the pattern's forward speed (Figure 3B). Forward speed varied in these trials, but only the sideways movements can produce a non-zero impulse response, and the sideways movements were identical. In other words, the sideslip perturbations are the only component of the flow field that is laterally asymmetrical, and so the only component that might cause differences in the left and right wing beat amplitudes (ΔWBA). We used paired *t*-tests to compare the peak responses of the medium speed pattern to the slower (*t* = 0.47, *P* = 0.63) and faster (*t* = 0.07, *P* = 0.95) patterns, and found no evidence that pattern speed had significant effects.

Next we wanted to determine if the relative forward speed of objects could affect the corrective response, even if absolute speed did not. We split the dots into two groups, some simulating forward motion with the m-sequence side-to-side modulation, others simulating a straight forward path (Figure 3C). The sideslipping dots, the ones that produce the dynamical response, moved identically in each trial, with a medium forward speed. The straight forward heading dots moved at one of the three speeds from the experiment above, slow, medium, or fast. Because of this the sideslipping dots were relatively faster, equal, or slower than the straight moving dots, but again, moving at the same absolute speed in each trial. We averaged the responses from 33 individual flies to estimate the linear filters in (Figure 3D). In this case the forward speed of the straight moving dots has a noticeable effect. When the sideslipping dots were the relatively slow, apparently distant objects, they produced a smaller response peak, and when they were the relatively fast, apparently near objects, the peak response was greater. With paired *t*-tests we compared the equal dot speed response to the relatively slow dot speed response (*t* = 1.75, *P* = 0.04) and the relatively fast dot speed response (*t* = 5.70, *P* < 0.0001).

### Following response to back and forth lateral motion

**Figure 4:**
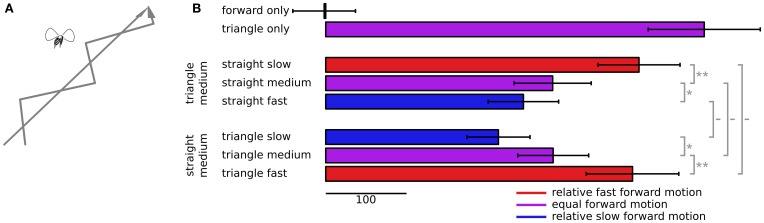
**Following behavior to dots slipping back and forth. (A)** The dots simulate two conflicting virtual paths for the fly, one straight forward and the other following a triangular waveform trajectory, forward motion plus alternating sideslip motion. **(B)** Cross correlations between the triangular waveform signal and ΔWBA, with error bars denoting standard error. The top two bars show correlations when the dots moved coherently, either all forward or all slipping back and forth in a triangular waveform pattern. The next three bars down show the correlations when the slipping triangular waveform dots advanced at a constant, intermediate forward speed, and the speed of the straight forward only moving dots varied between slower, the same, or faster than the slipping dots. The three bars on the bottom show the correlations when the straight forward moving dots advanced at a medium speed, and the triangular waveform slipping dots varied between slower, the same, or a faster speed. The sideways motion of the slipping dots remained the same in each case. The colors of each bar indicate the relative forward speed of the slipping dots, red for faster, blue for slower, and purple when the average dot speed was equal. The gray bars to the right show the results of paired *t*-tests, with symbols—for no significance, ^*^ for *P* < 0.05, ^**^ for *P* < 0.01.

A limitation of the white noise technique is that when dot trajectories indicate jittering and conflicting apparent paths, flies become reluctant to continue flying, and their wing beat responses drop dramatically or stop completely. Even with reasonably long m-sequences, estimates of the dynamical responses are noisy and difficult to distinguish, whereas coherent sideslip motion, modulated with white noise, can produce clean, repeatable estimates of an impulse response (Theobald et al., 2010a). To achieve more reliable measures of how strongly flies attend to different sets of dots we used a simpler stimulus of dots simulating forward motion, but slipping alternately left and right. This simulates a forward path that follows a triangular waveform (Figure 4A), which went through two left and right cycles and took just over 3.5 s. This didn't allow dynamical analysis of fly steering, but flies responded robustly compared to the jarring m-sequence, allowing us to test more combinations of relative dot speed. We tested 35 flies and used a cross-correlation with a 75 ms temporal delay, based on slip response delays measured in these and previous experiments, to produce a single measure of the strength of tracking response.

We presented two stimuli in isolation to determine the boundaries of tracking strength. The top two bars of Figure 4B show the responses to forward flowing dots alone, and to dots following a triangular forward waveform alone. The forward flowing dots have no lateral slip for the fly to track, and their correlation with a hypothetical lateral motion cannot statistically be distinguished from zero (*t* = 0.049, *P* = 0.48). On the other hand flies robustly track the alternating lateral slip of forward moving dots (*t* = 6.66, *P* < 0.0001).

We then presented mixtures of these two dot flow fields, but varied the forward speeds of each component. The next three bars in Figure 4B show the response correlations when the sideslipping dots advance at a medium speed, and the forward moving dots advance at a variable speed. When forward dots move at the same speed as the slipping dots (purple bar in the second group), the response is much less correlated to the slip than to the slipping dots alone (*t* = 3.53, *P* = 0.0006). But as with the peak responses in Figure 3, when the forward dots move faster (red bar in the second group), tracking becomes stronger (*t* = 3.06, *P* = 0.002), when they move slower (blue bar in the second group), tracking becomes weaker (*t* = 1.70, *P* = 0.05). The final bars show the converse experiment, in which we varied the speed of the slipping dots, and held the forward moving dots at a constant, middle speed. Here again, when the slipping dots were relatively slower (blue bar, third group), the tracking correlation was lower (*t* = 1.72, *P* = 0.05), and when the slipping dots were relatively faster (red bar, third group), the tracking correlation was higher (*t* = 2.08, *P* = 0.02).

The second and third groups of bars in Figure 4B are comparable, but the speeds were different. In the second group, the blue bar shows the responses to medium slipping dots paired with fast straight dots, which represents relatively slow motion of the slipping dots. But the blue bar in the third group shows the responses to dots moving at half the speed, slow slipping dots and medium straight forward moving dots, which is also relatively slow motion of the slipping dots. Even though the absolute forward speed of both groups of dots was half, the responses were not statistically distinguishable (*t* = 1.00, *P* = 0.16). The red bars show a similar situation. The bottom red bar shows responses to points that moved at twice the speed of the those that produced the upper red bar, but in both cases the slipping dots were the relatively faster, apparently nearer objects, and the responses again couldn't be distinguished statistically (*t* = 0.20, *P* = 0.42).

## Discussion

Even the largest insects possess still tiny brains to control their flight. To what degree they simply respond to stereotypical cues, and to what degree the somehow model the environment around them is an ongoing question. In some cases the aerial maneuvers insects perform may be simpler than they seem, but in other cases their brains may be capable of surprisingly complex processing. To examine one aspect of this processing, we measured flies' corrective responses as they appeared to stray from a forward heading according to visual features that, by motion parallax, were different distances away. Responses to apparently near and far features weren't equal in any of our experiments (our first hypothesis), even though the stimuli had the same brightness and size, and underwent the same lateral displacement. Lateral shifts from seemingly distant dots never produced a larger response than nearer dots (our third hypothesis), even though image motion from distant features signifies larger perturbations. Rather, our results supported our second hypothesis, that flies produce greater corrective responses to images whose forward flow indicates that they are nearer. Furthermore, over the range we tested, the absolute speed of forward optic flow has little effect on the corrective responses to sideways perturbations, but the relative forward speeds of individual features modulate the strength of corrections. Dots flowing at a medium forward speed produce a weaker corrective response when they slip to the side if they were mixed with faster moving dots, but a stronger response if they were mixed with slower moving dots. In no case is the response absolute, flies continue responding to dots that appear radially farther no matter how slow the relative speed. However the strength of response drops significantly when the relative speed decreases, either because perturbing dots slow down, or competing dots speed up. This is consistent with the interpretation that motion parallax, which is already known or suspected to be an important cue for many insect behaviors, also plays a part in the optomotor response to sideslip, and that fruit flies make use of depth cues when correcting unanticipated course deviations.

### Computational implications

Optomotor responses in flies were characterized early on with experiments in rotational drums, and indicated that flies steer to minimize the retinal slip in their frontal visual fields (Collett, 1980a,b; Mronz and Lehmann, 2008). This is a powerful technique to deliver wide-field visual stimulation and feedback, but limited to simulating rotation. An animal in nature both rotates and translates during flight, and these are surprisingly distinct operations. Rotation is a linear transformation, which preserves all angles between the viewer and surrounding features. Image speeds in the flow field are proportional to the viewer's angular speed. On the other hand translation is an affine transformation. Images expand from a focus in the direction of travel, recede to a focus directly behind, and move in between with variable speeds and dynamically changing angular relationships. These distinctions suggest there may be selective advantages when the nervous system analyzes rotational and translational flow fields differently.

In the fly brain, motion detection begins with a grid of correlation-type motion detectors, each responding to image displacement in a local region and preferred direction (Egelhaaf and Borst, 1993). But local motion cannot distinguish different types of self-motion. Tangential cells integrate responses over a swath of local detectors whose preferred directions match some specific pattern of optic flow (Krapp and Hengstenberg, 1996; Egelhaaf et al., 2002). These neurons function as matched filters to identify self-motion, such as advancing forward or perturbations to the side. This pooling effectively detects patterns of natural optic flow, but loses information about the speed of individual features. Rather, the response differences we measured here may be modulated by multiple processing pathways in the visual system. This is further evidenced by the result that in our tests flies never disregard perturbations, even if they appear distant, but modulate the strength of their responses.

### Visual ecology

Fruit flies in the wild fly forward in bouts interspersed by rapid turns (Tammero and Dickinson, 2002). This generates images on the retina that move from front to back, with relative speeds proportional to the object distance. But for a small flying insect, gusts and eddies of wind make it a challenge to keep a true heading. Flies, and any other target-seeking animal, must be able to compensate when they find themselves pushed off their intended course. In a natural setting flies will certainly rely on a variety of visual and mechanical cues to stabilize their flight (Sherman and Dickinson, 2004; Taylor and Krapp, 2008). Even considering just visual cues, there are several besides parallax that give hints about the three dimensional structure of the world. Truly distant objects will generally have smaller sizes and reduced contrast (Gibson, 1950). However, in our stimulus, we held size and brightness constant in order to eliminate them as potential cues. Additionally, distant objects appear to move more slowly and with smaller angular displacements during a sideslip event, just as they appear to move more slowly during forward translation. But we matched angular offsets for both groups of points as well. By matching the size, brightness, and sidways displacement of the slower dots to the faster ones, we might create the illusion that the slower dots, because they are far off, must be bigger and brighter objects in reality (but dimmed by their distance), and their displacement must signal a large deviation from course (only a large translation could generate so much image motion if the objects are far off). This led us to speculate early on that flies might respond *more* strongly to sideslip of the slower moving dots, to compensate for an apparently larger course error. However each test favored the alternative hypothesis, that flies attend more strongly to the faster moving, apparently nearer objects, measured by the magnitude of responses and strength of tracking. The slower moving, apparently farther objects always produced a response, but it was consistently weaker. This may be because nearer features are more relevant to an insect navigating outdoors. Of course, translational signals from near and far objects will generally correspond, and flies may be tuned to attend to nearer objects simply because they offer a larger, more reliable, estimate of course heading. Alternatively, far off objects may have some intrinsic benefits, like stability, that favors flies if they don't disregard them entirely.

Natural scenes are much more complex than the fields of dots we present in the cube. They contain edges, complex and overlapping shapes, and often a horizon. Our stimulus also omits color, polarized light, and the enormous range of brightness encountered in the wild. Finally, the tethered fly does not experience mechanical sensations that accompany real forward motion and perturbations. Given these considerations, it is remarkable that flies execute robust steering responses and closed loop bar fixation between trials. It implies that a tethered fly in a cube maintains, at least to some degree, the illusion of flying and controlling flight with differential wing beat amplitudes. The ΔWBA responses to each type of perturbation are unique (Theobald et al., 2010a), but we can't say with certainty what forces these differences generate during flight. Ideally flies might respond to sideslip deviation with an opposite sideslip response, but they may be unable to generate sideslip without coupling it to other forces and moments. Nonetheless, fruit flies can probably produce at least a few independent degrees of freedom in self-motion (Sugiura and Dickinson, 2009), and this allows a great deal of control. For example, a forward moving fly could compensate for a slip deviation with just yaw: if you imagine your car suddenly displaced to the wrong lane of traffic (translated to the side), unable to move sideways, you would (depending on the country you're in) steer left to get back to your lane (yaw), then right to adjust your heading and stay in that lane (another yaw). The optimal behavior depends on the environment and goal of flight, but even a few degrees of freedom in motion provides many options.

Flies execute remarkably fast maneuvers in response to visual events. Many insects, including flies, use parallax motion information as it is the most reliable depth cue available to them. Here we have shown that fruit flies integrate parallax information into their quick optomotor responses, which alters their corrective responses to otherwise identical sideslip perturbations. Further behavioral experiments, and potentially genetic screening, may help piece together the neural processing that allows this subtle change in response. It may be part of what enabled flies, with seemingly simple brains, to control the sophisticated and nuanced flight behavior that drove their tremendous evolutionary success.

### Conflict of interest statement

The authors declare that the research was conducted in the absence of any commercial or financial relationships that could be construed as a potential conflict of interest.
